# Resection of the Knee Joint

**Published:** 1859-05

**Authors:** 


### RESECTION OF THE KNEE JOINT.

[Discussion in the Society of Surgery, Paris, Nov. 3, 1858.]

M. Larrey presented, in behalf of Dr. Daniel Brainard, of Chicago, Illinois, a successful case of resection of the knee joint, and a table of many of the operations of this kind which have been performed up to this time.

On the occasion of the case communicated in the name of M. Brainard, M. Robert requested that the society should not judge the operation without knowledge of the final results of these operations. He asked if, after resection of the knee, anchylosis of the leg with the thigh, takes place; for, if not, it would be a very bad operation, one which he has no desire to perform.

M. Follin said, that M. Robert was already answered by facts, and we may affirm, that the immediate results are altogether as favorable as after amputation of the thigh in the continuity. The statistics which he had published, prove that osseous or strongly fibrous union generally takes place. The method of operating is not without its effect, and that of M. Ferguson appears to merit the preference. The operation ought to remain in the science, and the objection that the limb operated on does not grow, is not well founded, as a shoe with a high heel can supply the deficiency in point of length.

M. Broca thought that the deficiency of growth of the member is one of the gravest objections which can be urged against resection of the knee joint in young subjects. The want of solidity is not of frequent occurrence in the lower member.

M. Larrey, in speaking of a successful result, did not wish to pronounce finally upon resection of the knee. The failure to report unsuccessful cases renders statistics of operations necessarily incomplete. He thinks that resections of the kind can only be useful when bony union occurs.

M. Marjolin did not advocate the operation. Half the patients which he has seen operated died. The English are passionately fond of resections, but, considering the number of these operations performed at London, we might demand if some of the patients might not have been cured without any operation.

M. Giraldes could not agree with M. Marjolin. He had seen resections at London, which succeeded perfectly. The English surgeons had resolved the question of union. After this operation, the question of when it is proper to perform it (of indication) only remained to us to decide.

M. Maisonneune spoke against the operation.

M. Larrey reminded the society, that the English surgeons are not alone in favor of this operation. MM. Texter and Heyfelder, of Germany, had also performed it.

At the following meeting, M. Giraldes took up the subject again. He said that, from tables prepared by himself from the *Dublin Medical Journal*, the *Lancet*, the *Medical Times*, and *Edinburgh Monthly Journal*, it appears that the operation may be divided into four series :

| | | | | |
|---|---|---|---|---|
| 1, | from 1762 to 1830, | 19 | operations, . | 12 deaths. |
| 2, | " 1830 to 1854, | 31 | " . | 5 " |
| 3, | " 1854 to 1856, | 51 | " . | 9 " |
| 4, | " 1856 to 1858, | 26 | " . | 7 " |
| | Total, . | 127 | | 33 |

[NOTE.—We have, at the present time, knowledge of four other operations of resection of the knee joint, not yet, or only recently published. One of these is by Dr. J. M. Warren, for anchylosis; one by Dr. Burnham, of Lowell, also for anchylosis; one by Dr. J. W. Freer, of Chicago, for caries of the femur; one by Dr. Brainard, for the same cause. The first two of these seem to have been so far advanced as to justify the belief that a cure was effected. The latter was not as yet sufficiently advanced to determine that point; one having been performed about three months since, at the Mercy Hospital; the other, about eight weeks ago, at the U. S. Marine Hospital, Chicago. However, it may safely be affirmed of both these, that they are quite past the period of danger, and that they both seem in the way of recovery, with useful members.

We propose to return to this subject at no distant day. M. Giraldes has correctly stated it as one of indication only, as there can be no doubt of its value when properly employed. It is only by abuse that it can fall into discredit.

Since writing the above, we have noticed the report of two additional cases by M. Ferguson; both resulted fatally; one from disease of the lungs, the other from disease of the kidneys. If the surgeon were less distinguished and careful than M. Ferguson is known to be, we should be disposed to inquire if, in these cases, the operation was not contra-indicated.]

